# Robot-Assisted Bladder Neck Reconstruction in Refractory Vesicourethral Anastomotic Stenosis - A Single-Center Experience from a Specialized Urinary Tract Repair and Reconstruction Center

**DOI:** 10.1590/S1677-5538.IBJU.2025.0385

**Published:** 2025-09-30

**Authors:** Jianwen Huang, Changhao Hou, Song Li, Xiaoyong Hu, Ranxing Yang, Ying Wang, Nailong Cao, Jiong Zhang, Lujie Song, Qiang Fu

**Affiliations:** 1 Shanghai Sixth People's Hospital Affiliated to Shanghai Jiao Tong University School of Medicine Department of Urology Shanghai China Department of Urology, Shanghai Sixth People's Hospital Affiliated to Shanghai Jiao Tong University School of Medicine, Shanghai, China; 2 Shanghai Eastern Institute of Urologic Reconstruction Shanghai China Shanghai Eastern Institute of Urologic Reconstruction, Shanghai, China; 3 China RongTong Medical Healthcare Group Co.Ltd. Kaifeng 155 Hospital Department of Urology Kaifeng China Department of Urology, Kaifeng 155 Hospital, China RongTong Medical Healthcare Group Co.Ltd., Kaifeng, China

**Keywords:** Urinary Bladder, Robotic Surgical Procedures, Urinary Incontinence, Stress

## Abstract

**Objective:**

To investigate the efficacy and safety of robotic-assisted bladder neck reconstruction in patients with refractory vesicourethral anastomotic stenosis (VUAS) following radical prostatectomy (RP) or radical cystectomy (RC) with orthotopic neobladder (ONB) reconstruction.

**Methods:**

The clinical data from patients with VUAS who underwent robot-assisted bladder neck reconstruction at our center from August 2022 to February 2025 were retrospectively analyzed. The minimum postoperative follow-up period was 3 months, and bladder neck patency was defined as either the passage of a F16 flexible cystoscope or a maximum urinary flow rate (Qmax)>15 mL/s.

**Results:**

A total of 27 patients were analyzed, including 25 with a history of RP and 2 with a history of RC with ONB reconstruction. The median operative time was 210 min (interquartile range [IQR]:168-259), with a median estimated blood loss of 152 mL (IQR: 80–255) and a median postoperative hospital stay of 3.5 d (IQR: 3-6 d). At the median follow-up of 11 months (IQR: 3–34), 20 patients (74.1%) achieved patent reconstruction and 9 patients (75%) remained continent in 12 patients without preexisting stress urinary incontinence (SUI) at last follow-up. Postoperative complications occurred in five patients (18.5%), including two cases of Clavien-Dindo grade I and three cases of grade II.

**Conclusions:**

Robotic-assisted bladder neck reconstruction represents a safe and effective surgical option with high patency and low de novo SUI rates for refractory VUAS following RP or RC with ONB reconstruction.

## INTRODUCTION

Vesicourethral anastomotic stenosis (VUAS) resulting from scar formation at the vesicourethral anastomosis or neovesicourethral anastomosis, represents a rare yet severe complication following radical prostatectomy (RP) or radical cystectomy (RC) with orthotopic neobladder (ONB) reconstruction. The first-line treatment of VUAS is generally an endoluminal procedure, such as dilation, endoscopic incision, or resection (1, 2). However, in cases of recurrent failure or completely obliteration of the anastomosis, lower urinary tract reconstruction may be warranted for suitable candidates (1). The anatomical challenges of VUAS treatment cannot be overstated. The bladder neck's deep position within the narrow male pelvis complicates surgical access, while its proximity to the external urinary sphincter and rectum heightens risks of stress urinary incontinence (SUI) and rectal fistula formation. These factors collectively render VUAS one of the most technically demanding scenarios in lower urinary tract reconstruction (3).

Recent advances highlight robotic-assisted techniques as a promising alternative. Enhanced visualization and instrument maneuverability enable precise dissection and anastomotic reconstruction, potentially reducing complication rates (4-8). For non-obliterative VUAS, Y-V plasty with posterior urethral plate preservation has been described (5, 6). In obliterative cases, redo vesicourethral anastomosis - via retropubic, perineal, abdominoperineal, or robotic-assisted approaches is recommended after stenosis excision (1, 4). Notably, our team previously demonstrated the efficacy of Y-V plasty for refractory bladder neck contracture (BNC) following benign prostatic hyperplasia (BPH) surgery, with marked symptomatic relief (9). Despite these developments, robust evidence on robotic-assisted bladder neck reconstruction for refractory VUAS following RP or RC with ONB reconstruction remains limited. We hypothesized that robotic-assisted bladder neck reconstruction would provide a durable and effective treatment for these specific patients. This study therefore seeks to evaluate the outcomes of robotic-assisted bladder neck reconstruction in this specific patient population.

## MATERIALS AND METHODS

### Study design

This retrospective study included all patients who underwent robotic-assisted bladder neck reconstruction for VUAS following RP or RC with ONB reconstruction at our academic center from August 2022 to February 2025. Inclusion criteria were: (1) History of RP or RC with ONB; (2) Symptomatic patients with failure of at least two prior endoluminal treatments; (3) VUAS confirmed by cystourethrography or cystourethroscopy; Exclusion criteria included: (1) History of subtotal prostatectomy or BNC after BPH surgery; (2) Concomitant neurogenic bladder dysfunction. All patients underwent a 16Fr flexible cystoscopy (Olympus Europe, Hamburg, Germany). This examination was carried out to assess the diameter and location of the stenosis, the condition of the external urethral sphincter, and the presence of synchronous urethral stricture. Preoperatively, in addition to gathering demographic data, we also noted the patients’ history of pelvic radiation, pre-existing SUI, as well as the number and type of prior endoluminal VUAS treatments. This study was approved by the Ethics Committee of Shanghai Jiao Tong University Affiliated Sixth People's Hospital (No.2025-KY-178(K)). Moreover, all patients provided informed consent prior to their participation in the study.

### Surgical procedure

Robotic surgeries were carried out utilizing the Da Vinci Xi systems (Intuitive Surgical^®^). The patient was positioned supine, with the head tilted down and the feet elevated at an angle of 15–30°. A robotic - assisted surgical procedure was performed via an abdominal approach. Following the standard robotic ports placement, five ports were used at the umbilicus level. The peritoneum was then opened to expose the Retzius space, and the fat on the pelvic floor and bladder surface was carefully removed.

Traditional Y-V plasty: The technique was applied to cases of short and non-obliterative VUAS and has been detailed in previous publications (5,6,9). An inverted "Y" was marked on anterior aspect of the bladder neck using monopolar cautery to form an inverted "V" bladder flap. Subsequently, the inverted V-shaped flap was advanced and anastomosed to the tip of the stricture incision using barbed 3-0 suture, thereby widening the bladder neck.

**Figure 1:**
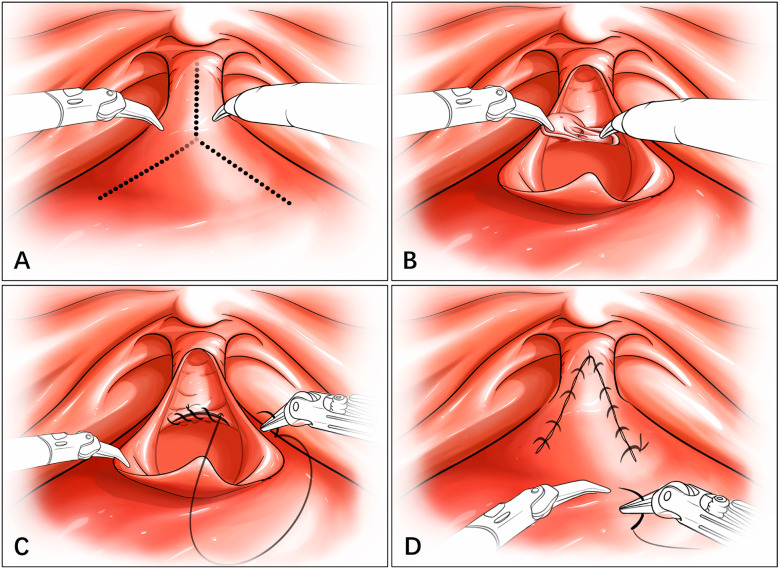
Schematic Diagram of Modified Y-V Plasty.

Modified Y-V plasty: This technology was applied to cases of short and completely obliterated VUAS. After the creation of an inverted "V" bladder flap, the scar tissue around the posterior plate of VUAS was dissected, Then, the posterior bladder neck mucosa was continuously sutured to the posterior urethral wall mucosa using 4-0 absorbable barbed sutures. The apex of the inverted V-shaped flap was anastomosed to the tip of the stricture incision using barbed 3-0 suture (Figure-1).

A) An inverted "Y" was marked on anterior aspect of the bladder neck; B) Scar tissue around the posterior plate of VUAS was dissected; C) Posterior bladder neck mucosa was continuously sutured to the posterior urethral wall mucosa; D) Apex of the inverted V-shaped flap was anastomosed to the tip of the stricture incision. (This figure was created by our team and has been previously published in Minerva Urology and Nephrology (23) with permission).

**Figure 2:**
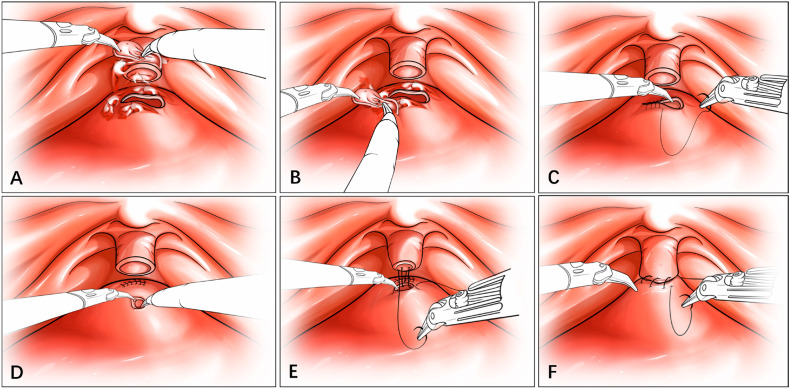
Schematic Diagram of Redo vesico-urethral anastomosis.

Redo vesico-urethral anastomosis (neo-bladder neck anastomosis): This technology was applied to cases of long and completely obliterated VUAS and has been described in previous publication (3, 4, 10). The obstructing fibrotic tissue was excised circumferentially. The native bladder neck was closed, and a neo-bladder neck was created. The bladder was rotated downwards, and the healthy neo-bladder neck was anastomosed to the urethral stump (Figure-2).

A) Resect the scar tissue around the distal anastomosis. B) Resect the scar tissue around the proximal anastomosis.; C) Native bladder neck was closed; D) Neo-bladder neck was created; E and F). Healthy neo-bladder neck was anastomosed to the urethral stump. (This figure was created by our team and has not been previously published).

A F18 three-lumen catheter was inserted. If necessary, a suprapubic catheter was placed simultaneously. The bladder mucosa was closed using barbed a 3-0 suture to ensure a leak-free closure. A drainage tube was placed, and the peritoneum was sutured with a barbed 2-0 suture to close the Retzius space.

### Postoperative follow-up/outcomes

Follow-up consisted of scheduled visits at 1, 3, 6 months and 1 year after surgery. At 1 month, the catheter was removed. Complications that emerged within 1 month postoperatively were collected and graded according to the Clavien–Dindo classification. Only patients with a minimum of 3 months of follow - up after the surgery were included in the study. At 3 months postoperatively, the patency and SUI were evaluated. Patency was defined as either the passage of a F16 flexible cystoscope or a maximum urinary flow rate (Qmax)>15 mL/s. SUI was defined as use of more than one pad per day postoperatively.

## RESULTS

This study involved 27 male patients. Among them, 25 (92.6%) had a history of RP, while 2 (7.4%) had a history of RC with ONB reconstruction. The demographic data are summarized in Table-1. Four patients (14.8%) had a history of pelvic radiation and 24 patients (88.9%) developed VUAS after robotic assisted RP (RA-RP), laparoscopic RP (L-RP) or RA-RC with ONB, and the remaining patients developed VUAS after open RP. The endoluminal treatment options for VUAS included dilation, endoscopic incision or resection. The median number of endoluminal treatments was 3. In addition, 12 patients (44.4%) did not have pre-existing SUI and 14 patients (51.9%) had a suprapubic catheter inserted preoperatively.

**Table 1:** Baseline demographic and preoperative data in patients with VUAS.

Patient demographics (n=27)		Results
Age (years), median (IQR)		65.8 (58-81)
BMI (kg/m^2^), median (IQR)		22.1 (17.5-29.6)
**History of surgery, n (%)**		
	RA-RP	16(59.2)
	L-RP	6(22.2)
	Open RP	3(11.1)
	RA-RC with ONB	2(7.4)
History of pelvic radiation, n (%)		4(14.8)
**Previous endoluminal treatment, n (%)**		
	Dilation	2(7.4)
	Incision/resection	12(44.4)
	Dilation + incision/resection	10(37.0)
	Incision/resection + antifibrotic drug	3(11.1)
Number of endoluminal treatment, median (IQR)		3 (2-7)
Preexisting SUI, n (%)		15(55.5)
**Bladder emptying method preoperatively, n (%)**		
	Spontaneous voiding	13(48.1)
	Suprapubic catheter	14(51.9)
**Synchronous disease, n (%)**		
	Anterior urethral stricture	1(3.7)
	Rectourethral fistula	1(3.7)
	Bladder stone	5 (18.5)

MI = Body Mass Index; RP = radical prostatectomy; RA-RP = robotic assisted radical prostatectomy; L-RP = laparoscopic radical prostatectomy; RC = radical cystectomy; ONB = orthotopic neobladder; SUI = stress urinary incontinence.

Perioperative information and postoperative data are shown in Table-2. In 13 (48.1%) patients, the VUAS were obliterated. The median length of VUAS was 1 cm (interquartile range [IQR]: 0.8-2.5). Regarding bladder neck reconstruction, 24 (88.9%) patients underwent Y-V plasty. Specifically, 14 patients received the traditional Y-V plasty, while 10 had the modified version. The remaining patients underwent redo vesico-urethral anastomosis. The median operative time was 210 minutes (IQR: 168-259). The median estimated blood loss was 152 mL (IQR: 80-255), and the median postoperative hospital stay was 3.5 days (IQR: 3-6 days). Two patients underwent ancillary procedures concomitantly with bladder neck reconstruction: one underwent urethroplasty for anterior urethral stricture and the other underwent repair for rectourethral fistula (RUF). A postoperative suprapubic catheter was used in 21 (77.8%) patients.

**Table 2:** Perioperative characteristics and postoperative complications according to Clavien-Dindo classification.

Parameter			Results
Patients(n)			27
**Stenosis characteristics**			
	Length(cm), median (IQR)		1.0(0.8-2.5)
	Non-obliterative, n (%)		14(51.9)
	Obliterative, n (%)		13 (48.1)
**Type of bladder neck reconstruction, n (%)**			
	Traditional Y-V plasty		14(51.9)
	Modified Y-V plasty		10(37.0)
	Redo vesico-urethral anastomosis		3(11.1)
Surgery time (minutes), median (IQR)			210(168-259)
Estimated blood loss (mL), median (IQR)			152(80–255)
Postoperative hospital Stay (days), median (IQR)			3.5(3-6)
**Ancillary procedures, n (%)**			2(7.4)
	Urethroplasty		1(3.7)
	Repair for RUF		1(3.7)
Catheterization time (days), median (IQR)			25 (21-30)
Postoperative suprapubic catheter, n (%)			21 (77.8)
Follow-up (month), median (IQR)			11(3–34)
**Postoperative complications within 1 month, n (%)**			5(18.5)
	Grade 1		2 (7.4)
	Grade 2		3 (11.1)
**Postoperative complications beyond 1 month, n (%)**			
	Grade 3b		1(3.7)
**Outcome of reconstruction, n (%)**			
	Patent		20(74.1)
	Recurrent		7(25.9)
***De novo* SUI, n (%)**			3(25.0)
	**Managed with**		
		AUS	2(16.7)
		Penis clamp	1(8.3)
**Recurrent VUAS managed with**			
	Intermittent endoluminal treatment		5(71.4)
	Permanent suprapubic catheter		2(28.6)

SUI = stress urinary incontinence; AUS = artificial urinary sphincter; RUF = rectourethral fistula

The median follow-up was 11 months (IQR: 3-34). Postoperative complications within 1 month postoperatively occurred in 5 patients (18.5%). Among them, 2 cases were classified as Clavien-Dindo grade I (1 case of transient hematuria required irrigation and 1 case of wound infection) and 3 cases were grade II (2 cases of serious abdominal infection and 1 case requiring blood transfusion). One patient with a history of pelvic radiation therapy developed a late postoperative complication: Although the vesicourethral anastomosis remained patent initially after conventional Y-V plasty, the patient developed a RUF at 10 months postoperatively, ultimately requiring diverting colostomy at 11 months.

Reconstruction was patent in 20 (74.1%) patients. Recurrent VUAS developed in 7 patients at a median of 3 months (IQR: 2-4) postoperatively. Among these 7 patients, 3 had a history of pelvic radiation after RP, 2 had a history of open RP, 1 had a history of ONB reconstruction and 1 had long and complete obliterated stenosis. For patients with recurrent VUAS, 5 patients underwent intermittent endoluminal treatment, of which two patients needed intermittent urethral dilation (every 3-6 months) to maintain patency, while the other three patients required intermittent endoscopic incision/resection (every 5-8 months) to maintain patency, and 2 patients used a permanent suprapubic catheter. At the last follow-up, 9 out of 12 patients (75%) without preexisting SUI remained continent. Three patients with de novo SUI had VUAS Involving the membranous urethra. Two of them underwent placement of artificial urinary sphincter (AUS) at 6 months postoperatively, and 1 used a penis clamp.

## DISCUSSION

Treating VUAS following RP or RC with ONB reconstruction presents greater challenges compared to BNC (2, 11). Patients with severe pelvic scar tissue and suboptimal tissue conditions, often resulting from prior surgery and pelvic radiation therapy, frequently encounter high recurrence rates and significant morbidity (4, 12). Moreover, after prostate removal, the bladder descends, the VUAS is located deeply, and the stenosis is in close proximity to the external urethral sphincter and the anterior rectal wall. This makes bladder neck reconstruction surgery difficult to expose and prone to damaging the external urethral sphincter and the anterior rectal wall. In this study, to enhance the success rate of VUAS treatment and reduce morbidity, we selected traditional Y-V plasty, modified Y-V plasty or redo vesico-urethral anastomosis to manage refractory VUAS, based on the severity of stenosis. We discovered that the transabdominal approach of robot-assisted bladder neck reconstruction for refractory VUAS was characterized by a high success rate and a low incidence of de novo SUI. This study is the first to demonstrate the successful application of a modified Y-V plasty for the treatment of VUAS. It is also the first to employ bladder neck reconstruction for successful management of VUAS following RC with ONB surgery.

The first-line treatments for VUAS after RP or RC with ONB mainly include dilation, endoscopic incision/resection, or intralesional injection of antifibrotic drugs (e.g, mitomycin C, triamcinolone, etc.) (2, 9, 13). An early study indicated that urethral dilation had a 59% success rate in treating VUAS (14). Hacker et al. reported successful outcomes of bladder neck incision combined with mitomycin C injection for VUAS treatment. The success rates were 45%, 64%, 82%, and 91% for one, two, three, and four treatments respectively (15). However, the limited number of cases and short follow-up in their study sparked considerable debate regarding the effectiveness of drug injections (15). Delchet et al. reported that the success rate of endoscopic treatment for VUAS after prostatectomy was 72.8%, which dropped to 62.9% after correcting for significant publication bias through a systematic review and meta-analysis (11). For VUAS occurring after RC with ONB, the success rate of each individual endoscopic treatment was 37%, and the use of adjuvant clean intermittent catheterization (CIC) improved the outcome of treatment (2). However, for patients who failed repeated endoscopic treatments or had completely obliterated stenosis, bladder neck reconstruction is a more suitable option. Currently, bladder neck reconstruction for VUAS mainly includes redo vesico-urethral anastomosis and bladder neck Y-V plasty (3-6,10). Studies have showed that redo anastomosis had a success rate of 60-91% in non-irradiated VUAS patients (1, 3-6, 16). However, this procedure requires excision of the scar tissue around the posterior wall of the anastomosis, increasing surgery complexity and the risk of rectal injury. In contrast, Y-V plasty only involves excision of the scar tissue around the anterior wall of the anastomosis, followed by suturing the inverted V-shaped anterior bladder flap to distal urethra (6). Therefore, several authors believe that bladder neck Y-V plasty may be more appropriate for short or non-obliterative VUAS cases (3, 6, 17). In cases of long or completely obliterated stenosis, traditional Y-V plasty may not prevent scar tissue from the posterior wall of the bladder neck from affecting the patency of the anastomosis. In this study, we applied traditional Y-V plasty to 14 cases of short and non-obliterative VUAS, modified Y-V plasty to 10 cases of short and completely obliterated VUAS, and redo vesico-urethral anastomosis to 3 cases of long and completely obliterated VUAS. We described a modified Y-V plasty technique where the scar tissue around the posterior wall of the bladder neck was first excised, followed by continuous suturing of the posterior wall of bladder neck with the posterior wall of urethral stump, and then the inverted V-shaped flap was anastomosed to the urethral stump. For long and complete obliterated stenosis, suturing posterior wall of the bladder neck to the distal urethra can be difficult due to severe scar tissue around the bladder neck and poor mobility of the posterior bladder neck. Additionally, the long stenosis is closer to the external urethral sphincter, and Y-V plasty requires a longitudinal incision of the anterior wall of the normal urethra, which inevitably increases the risk of external urethral sphincter injury. In such cases, we used a redo vesico-urethral anastomosis (neo-bladder neck anastomosis) technique. In this technique, the obstructing fibrotic tissue was circumferentially excised, the native bladder neck was closed, a neo-bladder neck was created, the bladder was rotated downwards, and the healthy neo-bladder neck was anastomosed to the urethral stump to ensure mucosal apposition and a tension-free anastomosis (4, 10).

In the study, a rigorous analysis of recurrence risk factors was unproductive due to the low number of VUAS recurrence cases. Previous radiation therapy has been identified as a risk factor for VUAS treatment failure (4, 16, 18). Moreover, radiation-induced bladder toxicity may cause urethral necrosis, leading to the failure of VUAS reconstruction (18, 19). In this study, recurrence cases were mainly found in patients with a history of pelvic radiotherapy, open RP, ONB, and long and completely obliterated VUAS. Pelvic radiotherapy can lead to poor tissue conditions of the bladder wall flap and urethra, while ileal-reconstructed neobladder may result in poor tissue condition of the neobladder wall flap due to inadequate mesenteric blood supply. Thus, radiotherapy and orthotopic neobladder may increase the risk of VUAS recurrence. In such cases, using rectus abdominis, gracilis, peritoneum, or omentum flap coverage may improve the tissue conditions for bladder neck construction and enhance surgery success rate (3, 20). Additionally, in cases with a history of open surgery and long and completely obliterated VUAS, there is a large amount of scar tissue around the pelvic anastomosis, and a transabdominal approach alone may not ensure a tension-free anastomosis of the bladder wall flap or bladder neck with the distal urethra. Therefore, a combined transabdominal-perineal approach may reduce anastomosis tension and increase the procedure's success rate.

Surgical approaches for bladder neck reconstruction include the perineal, abdominal, or combined perineal-abdominal approach (1). Studies have showed that bladder neck reconstruction via perineal approach inevitably leads to postoperative urinary incontinence as it disrupts the external urethral sphincter (6, 16, 18). Pfalzgraf et al. reported that bladder neck reconstruction via abdominal route resulted in a 36% (4/11) incidence of de novo urinary incontinence in VUAS patients (21). Nikolavsky et al. who mainly used an abdominal approach for bladder neck reconstruction, reported a 58% incidence of postoperative incontinence (20). Kirshenbaum et al. reported on robotic-assisted abdominal route bladder neck reconstruction in five VUAS patients, none of whom experienced postoperative urinary incontinence (6). Therefore, some researchers suggest that the abdominal approach is preferable for patients with good preoperative urinary continence, although the two approaches have never been directly compared in terms of urinary continence (6, 20). Additionally, the abdominal approach avoids disrupting the perineal anatomy and blood supply, making subsequent AUS implantation less complicated (6). In this study, all VUAS patients were treated via the abdominal approach. We found that 9 (75%) out of 12 patients without preexisting SUI achieved complete continence within 1 to 12 months postoperatively, while 3 (25%) patients developed de novo SUI. This outcome was superior to the 64% reported by Pfalzgraf et al. but inferior to the 85% reported by Shakir et al. (4, 21).

Rectal injury is one of the most concerning complications after redo vesico-urethral anastomosis. By utilizing visualization techniques such as suction device assistance, rectal manipulation, and image guidance with near-infrared fluorescence (NIRF) imaging and/or transrectal ultrasound (TRUS), it is possible to more precisely dissect and excise behind the bladder neck, thus avoiding rectal injury (22). Urine leakage can cause anastomotic failure, restenosis, and/or infection. There have been reports of cases where urine leakage or infection led to a fistula to the pubic symphysis, resulting in osteomyelitis, which may require subsequent pubectomy (6). The best way to prevent this complication is to ensure a well-vascularized and tension-free anastomosis.

Our study has several limitations. Firstly, the limited number of patients is a major drawback. Secondly, the relatively short follow-up period for patients undergoing robotic-assisted bladder neck reconstruction for refractory VUAS is also a limitation. Larger scale prospective studies with longer follow-up periods are needed. Additionally, these data represent our learning curve, and the outcomes may improve with increasing experience. Finally, all surgeries in this study were performed by a single highly experienced robotic-assisted surgeon, so the results cannot be generalized to all clinical centers.

## CONCLUSIONS

Robotic-assisted bladder neck reconstruction via an abdominal approach is a safe and effective surgical option with high patency and low de novo SUI rates for refractory VUAS following RP or RC with ONB reconstruction.

## Data Availability

Uninformed

## ABBREVIATIONS

VUAS: vesicourethral anastomotic stenosis
RP: radical prostatectomy
RC: radical cystectomy
ONB: orthotopic neobladder
IQR: interquartile range
Qmax: maximum urinary flow rate
SUI: stress urinary incontinence
BNC: bladder neck contracture
BPH: benign prostatic hyperplasia
L-RP: robotic assisted RP (RA-RP), laparoscopic RP
RUF: rectourethral fistula
CIC: clean intermittent catheterization
NIRF: near-infrared fluorescence
TRUS: transrectal ultrasound
